# Magnitude of Incapability and Pain Intensity are Associated More with Unhelpful Thoughts Than Stressful Life Events

**DOI:** 10.1177/24705470231179644

**Published:** 2023-06-08

**Authors:** Melle M Broekman, Niels Brinkman, Sina Ramtin, Marielle Ngoue, David Ring, Prakash Jayakumar

**Affiliations:** Department of Surgery and Perioperative Care, 377659 Dell Medical School at the University of Texas at Austin, Austin, TX, USA

**Keywords:** Social health, childhood events, difficult life events, capability, pain intensity

## Abstract

Prior studies show that stressful life events are associated with greater magnitude of incapability and symptom intensity. We sought to understand the association of such events (i.e., both adverse childhood experiences *and* recent difficult life events [DLEs]) alongside feelings of worry or despair and unhelpful, on the magnitude of incapability and symptom intensity in musculoskeletal patients. One hundred and thirty-six patients presenting for musculoskeletal specialty care completed measures of incapability, pain intensity, adverse childhood experiences, DLEs in the last year, unhelpful thoughts, symptoms of anxiety and depression, and sociodemographic factors. Factors associated with the magnitude of incapability and pain intensity were sought in multivariable analysis. Accounting for potential confounders, greater incapability was associated with greater unhelpful thoughts (RC  =  −0.81; 95% CI  =  −1.2 to −0.42; *P*  ≤  .001), but not with stressful life events (either during childhood or more recently). Greater pain intensity was associated with greater unhelpful thoughts(RC  =  0.25; 95% CI  =  0.16 to 0.35; *P*  ≤  .001) and being divorced or widowed (RC  =  1.8; 96% CI  =  0.43 to 3.2; *P*  =  .011), but again, not with stressful life events. The strong association of unhelpful thoughts with magnitude of incapability and pain intensity can motivate musculoskeletal specialists to anticipate patients expressing negative pain thoughts and behaviors. Future studies might account for social and environmental context behind stressful life events and the influence of resiliency and pain-coping strategies on these interactions.

**Level of Evidence:**

Level III, prognostic study

## Introduction

Recent difficult life events (DLEs) can be a source of ongoing feelings of worry and despair and are associated with greater incapability.^1,2^ Studies have also shown that stressful events experienced during childhood (or adverse childhood events [ACEs]) bear longer-term consequences on physical and emotional health and wellbeing, including one's thoughts, behaviors, and adaptive response to pain.^3,7^ A study using Canadian Community Health Survey Data found that mental health and socioeconomic status mediated the degree to which ACEs were associated with a wide range of chronic illnesses in adulthood including musculoskeletal conditions.^8^ On one hand, experiencing ACEs may perpetuate further stressful experiences extending into adulthood, while on the other, such events can serve to catalyze the development of resilience and effective coping strategies that may benefit long-term biopsychosocial health.^9^ Either way, mental and social health support may buffer and bolster the experience of adverse events.

There is also an expanding body of evidence for the dominant association between feelings of worry or despair and unhelpful thoughts on the magnitude of incapability and symptom intensity.^10,17^ We sought to understand the interplay of the impact of stressful life events (both ACEs and recent DLEs) alongside feelings of negative mood and negative pain thoughts, on the magnitude of incapability and pain intensity (measured using patient-reported outcome measures [PROMs]) in a musculoskeletal population.

This study assesses two questions: (1) What are the associations of magnitude of incapability with stressful adverse childhood experiences, a difficult life event in the last year, symptoms of anxiety or depression, and unhelpful thoughts in people with musculoskeletal conditions? (2) What are the associations of pain intensity with stressful adverse childhood experiences, a difficult life event in the last year, symptoms of anxiety or depression, and unhelpful thoughts in people with musculoskeletal conditions? The findings may help prioritize and select mental and social health opportunities in relation to stressful life experiences and psychosocial factors.

## Patients and Methods

### Study Design and Setting

In an Institutional Review Board (IRB)-approved, cross-sectional study, patients with musculoskeletal illness who presented to one of several musculoskeletal specialist outpatient offices in an urban area of the United States were invited to participate by a research assistant who was not directly involved in their care. All musculoskeletal injuries were eligible for this study. All patients were English- or Spanish-speaking. Participants were excluded if they were younger than 18 years old or had cognitive dysfunction. Patients were asked to complete the survey before or after the visit with the specialist in a private examination room. All patients gave verbal consent before collection of any data. Survey completion by patients was accepted by the IRB as equivalent to written informed consent. All surveys were completed on a tablet using Health Insurance and Portability Accounting Act-compliant software platform (Research Electronic Data Capture [REDCap], Vanderbilt, TN). Participants completed surveys measuring the magnitude of incapability, pain intensity, ACEs, DLEs in the last year, negative pain thoughts, and sociodemographic factors (age, sex, annual household income, BMI, race, marital status, highest level education, work status, and insurance status).

### Participants

A total of 203 eligible participants started the surveys with 136 (67%) completing them fully. Participants with incomplete survey responses were excluded. Incomplete surveys were due to two main reasons: First, due to the COVID-19 pandemic and minimizing the risk of transmission, patients were invited to complete surveys using their phones rather than office-based tablet devices. The opportunity to complete surveys in their own time rather than in clinic where the tablet would have to be returned, increased the risk of people leaving the office and only partially completing the survey if at all. Second, a temporary malfunction in REDCap during the study led to the survey being automatically shut down before completion. During the study, we introduced the use of tablet devices and a decontamination protocol between patients and the malfunction was rectified. We continued to enroll patients until we had a sufficient number of complete survey responses.

One hundred and thirty-six patients with completed surveys were used for analysis. Forty-nine percent (n  =  67) were men. The mean age was 56 years  ±  16 years and 36% (n  =  49) had an annual household income of greater than $121,000 (Table 1).

**Table 1.** Demographics of Cohort.

**Variables**	**Value** ^a^
N	136
Age	56 ± 16
Male	67 (49%)
Smoker	8 (5.9%)
Body mass index	28 ± 6.3
Race	
White	99 (73%)
African-American	9 (6.6%)
Latino/Hispanic	22 (16%)
Asian	3 (2.2%)
Other	3 (2.2%)
Marital status	
Married/partner	87 (64%)
Divorced/seperated	12 (8.8%)
Single	31 (23%)
Widowed	6 (4.4%)
Highest level of education	
Elementary school	1 (0.74%)
High school	28 (21%)
2-year college	26 (19%)
4-year college	44 (32%)
Post-college graduate degree (e.g., PhD)	37 (27%)
Work status	
Employed	72 (53%)
Unemployed	11 (8.1%)
Disabled	4 (2.9%)
Retired	43 (32%)
Other (e.g., student, homemaker, etc.)	6 (4.4%)
Annual household income	
<$24,000	24 (18%)
$24,000–$46,000	15 (11%)
$46,000–$75,000	19 (14%)
$75,000–$121,000	29 (21%)
>$121,000	49 (36%)
Insurance status	
Medicare	47 (35%)
Medicaid	4 (2.9%)
Private	56 (41%)
Uninsured	5 (3.7%)
Other	24 (18%)
Pain	5 (2–7)
Adverse childhood events (N)	1 (0–2.5)
Difficult live events	
Number of events	4.5 (2–8)
Holmes–Rahe score	119 (44–195)
PROMIS Anxiety CAT	53 ± 9.9
PROMIS Depression CAT	50 ± 9.3
PROMIS Physical Function CAT	44 ± 9.8
NPTQ-4	7 (4–11)

Abbreviations: NPTQ-4, 4-question negative pain thoughts questionnaire.

^a^ Value is displayed as median with interquartile range for continuous nonparametric variables, as mean with standard deviation for continuous variables with normal distribution, and as number with percentage for categorial variables.

### Measurements

We measured magnitude of incapability using the Patient Reported Outcome Measurement Information System Physical Function Computerized Adaptive Test (PROMIS PF CAT).^18,19^ PROMIS CATs are adaptive questionnaires designed to assess a range of health domains with new questions administered based on the participant's last response. PROMIS questionnaires are scaled to the general population living in the United States of America. A score of 50 represents the average score in this population, with one SD being represented by 10 points above or below 50. Lower PROMIS PF scores indicate greater incapability.

We measured mental health using PROMIS anxiety CAT to measure symptoms of anxiety, PROMIS depression CAT to measure symptoms of depression, and a 4-question negative pain thoughts questionnaire (NPTQ4) to quantify unhelpful thinking.^20,21^ The NPTQ4 measures unhelpful thoughts in response to nociception using 4 items, each scored on a 6-point Likert scale. Higher PROMIS Anxiety and PROMIS Depression scores respectively indicate greater symptoms of anxiety and depression. Higher NPTQ4 scores indicate greater unhelpful thoughts.

Pain intensity was measured using a Numeric Rating Scale for Pain, an 11-point ordinal scale from 0 representing no pain to 10, the worst pain imaginable.^22^

We measured adverse childhood experiences (ACE) using the ACE scale, which is a validated 10-item binary questionnaire (answered with “yes” or “no”) measuring domains of physical, emotional, and sexual abuse before the age of 18.^23^ Scores vary from 0 to 10 with higher scores representing more adverse childhood experiences.^24^ The median (IQR) number of DLEs was 4.5 (IQR  =  2 to 8) and for ACEs 1.0 (IQR  =  0–2.5) (Table 1). More ACEs were associated with more DLEs (spearman rho  =  0.40; *P* < 0.001).

Finally, we used a list of DLEs based on the Holmes–Rahe stress inventory to count DLEs within the past year: death of spouse, marital separation, divorce, detention in jail or other institution, death of a close family member, major personal injury or illness, being fired at work, major decrease in the health or behavior of a family member, eviction or major damage to one's home, and being a victim of a crime.^25^ We also used the Holmes–Rahe stress inventory weighted score, where every included life event is given a specific weighting to account for the relative impact it may have on a person.^26^ Higher scores represent more impact of DLEs.

### Ethical Approval

We obtained local institutional review board approval for this cross-sectional study (Registration No STUDY00001581).

### Statistical Analysis

We performed descriptive statistics where continuous data with normal distribution were described as mean with standard deviation (SD), continuous nonparametric data were described as median with interquartile range (IQR), and categorical data was described in numbers with percentage. Student *t*-test and one-way analysis of variance (ANOVA) were used for continuous data with a normal distribution, and Mann–Whitney U tests and Kruskal–Wallis H tests were used for nonparametric continuous data. The Spearman correlation coefficient was calculated to assess the correlation between all continuous explanatory variables and (a) PROMIS PF CAT and (b) NRS pain intensity score. All variables with a *P* value below .10 in bivariate analysis were moved to multivariable analysis to limit the number of explanatory variables in order to have at least 10 observations per included variable (Table 2).^27^ Two separate multiple linear regression models were used to seek factors associated with PROMIS PF CAT and NRS pain intensity score. All variables with a *P* value below .05 were considered statistically significant.

**Table 2.** Bivariate Analysis of Factors Associated With PROMIS Physical Function CAT and Numeric Rating Scale for Pain.

	Promis Physical Function Cat^a^		Numeric Rating Scale (PAIN)	
Variables	Mean ± SD	*P*-Value	Median (IQR)	*P*-Value
Gender		.44		.61
Male	44 ± 10		5 (2–7)	
Female	43 ± 9.4		5 (3–7)	
Smoker		.65		.72
Yes	42 ± 13		5 (3–6.5)	
No	44 ± 9.6		5 (2–7)	
Race		.050		.045
White	45 ± 9.7		4 (2–6)	
Latino/Hispanic	41 ± 9.6		5 (3–8)	
Other	39 ± 9.1		5 (5–8)	
Marital status		.055		.0027
Married/partner	44 ± 10		5 (2–7)	
Divorced/widowed	38 ± 8.5		6.5 (5–8)	
Single	45 ± 9.2		4 (2–6)	
Highest level of education		.097		<.001
High school and less	42 ± 9.6		5 (4–8)	
2-year college	41 ± 8.2		6 (5–8)	
4-year college	46 ± 10		3 (1–5)	
Post-college graduate degree (e.g., PhD)	44 ± 9.7		4 (2–5)	
Work status		.027		.0052
Employed	46 ± 11		4 (1.5–5.5)	
Unemployed/disabled	38 ± 8.0		7 (5–8)	
Retired	43 ± 8.6		5 (3–7)	
Other (e.g., student, homemaker, etc.)	40 ± 7.1		7 (5–8)	
Annual household income		.0041		.0021
<$24,000	39 ± 8.4		7 (5–8)	
$24,000–$46,000	40 ± 9.2		5 (5–8)	
$46,000–$75,000	42 ± 9.2		5 (3–7)	
$75,000–$121,000	44 ± 8.0		4 (2–5)	
>$121,000	47 ± 11		3 (1–6)	
Insurance status		.0069		.0059
Medicare	42 ± 7.9		5 (3–7)	
Medicaid/Other	41 ± 8.8		5 (3–8)	
Private	47 ± 11		3.5 (1–5.5)	
	Correlation coefficient (ρ)		Correlation coefficient (ρ)	
Age	−0.11	0.21	0.13	0.13
Body mass index	−0.17	0.060	0.25	0.0039
Adverse childhood events	−0.074	0.39	0.072	0.41
Difficult life events				
Number of events	−0.25	0.003	0.14	0.11
Holmes–Rahe score	−0.30	<0.001	0.20	0.019
PROMIS Anxiety CAT^b^	−0.32	<0.001	0.23	0.0067
PROMIS Depression CAT^b^	−0.33	<0.001	0.15	0.073
NPTQ-4	−0.55	<0.001	0.52	<0.001

Abbreviations: NPTQ-4  =  4-question negative pain thoughts questionnaire.

^a^ Value is displayed as median with interquartile range for continuous nonparametric variables, as mean with standard deviation for continuous variables with normal distribution. Spearman correlation indicated by ρ. The variables race, marital status, highest level of education, work status, and insurance status were pooled due to low numbers. All variables with *P* < .10 were moved to multivariable analysis.

^b^ PROMIS anxiety and PROMIS depression were removed from the multivariable regression model due to multicollinearity.

Due to multicollinearity (intercorrelation) between the mental health variables (Appendix 1), we decided to run multiple models with one mental health variable at the time and to only include the model that had the best model fit (lowest Akaike Information Criterion).^28^ The model with NPTQ-4 had the best fit and was selected, and other mental health variables were excluded. ACEs were not moved to multivariable analysis since the *P* value was above the .10 threshold in bivariate analysis.

An a priori sample size calculation determined that 136 patients would generate 80% statistical power based on a multivariable linear regression model with 8 explanatory variables, if DLEs account for 10% of the variation in PROMIS PF, and if the full model accounts for 25% of the overall variation, with alpha set at 0.05.

Statistical analyses were performed in STATA (STATACorp, College Station, Texas, USA).

## Results

Question 1: What are the associations of magnitude of incapability with stressful adverse childhood experiences, a difficult life event in the last year, symptoms of anxiety or depression, and unhelpful thoughts in people with musculoskeletal conditions?

Accounting for potential confounders such as marital status, employment status, and annual household income, greater incapability was associated with greater unhelpful thinking (Regression coefficient [RC]  =  −0.82; 95% confidence interval [CI]  =  −1.2 to −0.42]; *P*  ≤  .001), but not with a higher Holmes–Rahe score (Table 3).

Question 2 What are the associations of pain intensity with stressful adverse childhood experiences, a difficult life event in the last year, symptoms of anxiety or depression, and unhelpful thoughts in people with musculoskeletal conditions?

**Table 3.** Multiple Linear Regression Analysis of Factors Associated With PROMIS Physical Function CAT.

	Regression Coefficient (95% Confidence Interval)	Standard Error	*P*-Value	Partial *R*^2^	*R* ^2^	Adjusted *R*^2^
			** **		0.40	0.27
Body mass index	−0.010 (−0.29 to 0.27)	0.14	.94	0.000046		
Race						
White	Reference value					
Latino/Hispanic	−1.8 (−7.4 to 3.7)	2.8	.51	0.004		
Other	−4.5 (−10 to 1.3)	3.0	.13	0021		
Marital status						
Married/partner	Reference value					
Divorced/Widowed	−1.6 (−7.1 to 4.0)	2.8	.58	0.0029		
Single	1.7 (−3.1 to 6.5)	2.4	.49	0.0044		
Highest level of education						
High school and less	Reference value					
2-year college	−3.5 (−8.9 to 2.0)	2.8	.21	0.014		
4-year college	−0.61 (−5.9 to 4.7)	2.7	.82	0.00049		
Post-college graduate degree (e.g., PhD)	−3.8 (−9.2 to 1.6)	2.7	.17	0.018		
Work status						
Employed	Reference value					
Unemployed	−2.5 (−9.1 to 4.0)	3.3	.44	0.0055		
Retired	−2.2 (−6.7 to 2.2)	2.3	.32	0.0091		
Other (e.g., student, homemaker, etc.)	−5.3 (−14 to 3.0)	4.2	.21	0.015		
Annual household income						
<$24,000	Reference value					
$24,000–$46,000	−1.3 (−8.2 to 5.5)	3.5	.71	0.0013		
$46,000–$75,000	0.85 (−6.5 to 8.2)	3.7	.82	0.00049		
$75,000–$121,000	0.64 (−7.0 to 8.3)	3.9	.87	0.00026		
>$121,000	3.5 (−4.4 to 11)	4.0	.39	0.007		
Insurance status						
Medicare	Reference value					
Medicaid/Other	4.0 (−1.9 to 9.9)	3.0	.19	0.016		
Private	2.1 (−2.7 to 6.8)	2.4	.39	0.0068		
Holmes–Rahe score (Difficult Live Events)^a^	−0.01 (−0.026 to 0.0050)	0.077	.18	0.016		
NPTQ-4	−0.81 (−1.2 to −0.42)	0.19	**<**.**001**	0.14		

Bold indicates statistical significance, *P* < .05.

Abbreviations: NPTQ-4  =  4-question negative pain thoughts questionnaire.

^a^ PROMIS anxiety and PROMIS depression were removed from the multivariable regression model due to multicollinearity.

Accounting for potential confounders such as marital status, employment status and annual household income in multivariable analysis, greater pain intensity was associated with greater unhelpful thoughts (RC  =  0.25; 95% CI  =  0.16 to 0.35; *P*  ≤  .001) and a divorced or widowed marital status (RC  =  0.44; 96% CI  =  0.12 to 0.75; *P*  =  .007), but not with the Holmes–Rahe score (Table 4).

**Table 4.** Multiple Linear Regression Analysis of Factors Associated With Numeric Rating Scale for Pain Intensity.

	Regression Coefficient (95% Confidence Interval)	Standard Error	*P*-Value	Partial *R*^2^	*R* ^2^	Adjusted *R*^2^
			** **		0.48	0.39
Body mass index	0.051 (−0.020 to 0.12)	0.036	.16	0.018		
Race						
White	Reference value					
Latino/Hispanic	−0.13 (−1.5 to 1.3)	0.70	.86	0.0003		
Other	0.60 (−0.87 to 2.1)	0.74	.42	0.006		
Marital status						
Married/partner	Reference value					
Divorced/widowed	1.8 (0.43 to 3.2)	0.70	.**011**	0.059		
Single	0.14 (−1.1 to 1.3)	0.61	.82	0.00047		
Highest level of education						
High school and less	Reference value					
2-year college	0.46 (−0.91 to 1.8)	0.69	.51	0.0041		
4-year college	−1.0 (−3.4 to 0.30)	0.67	.13	0.021		
Post-college graduate degree (e.g., PhD)	−0.86 (−2.2 to 0.50)	0.68	.22	0.014		
Work status						
Employed	Reference value					
Unemployed	1.4 (−0.28 to 3.0)	0.83	.10	0.024		
Retired	0.20 (−0.93 to 1.3)	0.57	.73	0.0011		
Other (e.g., student, homemaker, etc.)	1.2 (−0.88 to 3.3)	1.1	.26	0.012		
Annual household income						
<$24,000	Reference value					
$24,000–$46,000	1.7 (−0.052 to 3.4)	0.87	.057	0.033		
$46,000–$75,000	0.66 (−1.2 to 2.5)	0.93	.48	0.0046		
$75,000–$121,000	0.67 (−1.3 to 2.6)	0.97	.49	0.0044		
>$121,000	1.2 (−0.77 to 3.2)	1.0	.23	0.013		
Insurance status						
Medicare	Reference value					
Medicaid/Other	−1.5 (−3.0 to 0.0068)	0.75	.051	0.035		
Private	−0.98 (−2.2 to 0.20)	0.60	.10	0.024		
Holmes–Rahe Score (Difficult Live Events)^a^	0.00031 (−0.0035 to 0.0042)	0.0019	.88	0.00023		
NPTQ-4	0.25 (0.16 to 0.35)	0.049	**<**.**001**	0.20		

Bold indicates statistical significance, *P* < .05.

Abbreviations: NPTQ-4  =  4-question negative pain thoughts questionnaire.

^a^ PROMIS anxiety and PROMIS depression were removed from the multivariable regression model due to multicollinearity.

## Discussion

There is growing interest in the relationship between symptom intensity and level of incapability (PROMs) with psychosocial determinants of health in musculoskeletal populations, including stressful life events such as ACEs and DLEs.^3,16,17,29,30^ Our study showed unhelpful thinking was more dominantly associated with PROMs than stressful life events.

### Limitations

There were several limitations to this study. First, our study focuses on the interplay between stressful life events and PROMs in a population with musculoskeletal illness ie, people who felt their symptomatic concerns were sufficient to seek musculoskeletal specialty care. We recognize that our findings might differ if the study was conducted in a general population. Second, the number of adverse childhood experiences was limited in our cohort that was relatively economically advantaged (57% of patients had an annual household income greater than $75,000). It is possible that there would be a higher occurrence of ACEs in a less privileged cohort, which might alter the results. Studies involving different populations, may spotlight variations in worry, despair, and unhelpful thoughts among different racial and ethnic groups as well as their thoughts and behaviors regarding stressful life events. For instance, American Indians are documented to experience more DLEs and distress, which were associated with greater pain-related symptoms of anxiety and unhelpful thinking (pain catastrophizing).^31^ Third, 33% of all patients who started the survey did not complete questionnaires and were thus excluded from the study. Although one reason was the technical malfunction on REDCap, which could not be anticipated, future studies might achieve improved completion rates by encouraging patients to complete surveys while in clinic, incentivizing patients, and providing them the option of survey administration via email, telephone call, or text. Fourth, we did not register diagnoses. All patients with any type of musculoskeletal illness were eligible for this study. The distribution of the diagnoses should be representative of musculoskeletal specialty care. Finally, we did not have data on whether the patient completed their surveys before or after the clinician visit, however, our studies have found that one visit does not typical alter questionnaire scores.^32^

### Association Between Magnitude of Incapability and Stressful Life Events

Unhelpful thoughts may be more important than the recollection of stressful life events (ACEs and DLEs) in the association with one's level of incapability. Perhaps the recollection of stressful life events (which may be easier when events are more recent) is only impactful to the extent that it reinforces unhelpful thinking—a factor that is strongly associated with the magnitude of incapability.^33,34^ This may explain why there was some level of association between recent DLEs, rather than ACEs, and magnitude of incapability (seen in bivariate analysis). Adults experiencing the effects of DLEs, including symptoms of posttraumatic stress disorder (involving intrusion, avoidance, and hyperarousal), are shown to report higher levels of pain intensity, pain bothersomeness, greater limitations, and worse mental health (worry and despair, catastrophic thinking, and lower self-efficacy).^2,35,42^

In our study, ACEs were not associated with the magnitude of incapability. Another study also shows that ACEs do not correlate with the magnitude of incapability in adults with musculoskeletal illness, unless such events are accompanied by greater symptoms of anxiety or less effective coping strategies.^9^ ACEs are known risk factors for developing emotional concerns (e.g., symptoms of anxiety and depression, excessive fear), negative addictive health behaviors (e.g., nicotine use or dependence on illicit substances), and worse impairment, cognitive function, and chronic conditions across the lifespan into adulthood.^43,44^ Such sequelae may occur within the backdrop of a variety of contextual (environmental and personal) factors ranging from parental loss or divorce, childhood accidental trauma or nonaccidental trauma, financial hardships, hospitalizations and surgery, and social unmet needs (lack of basic resources such as food, safe housing, and transportation).^5,45^

Notably, stressful life events in general may stimulate growth, adaptation, and building resilience in the face of adversity.^3,46^ Cumulative lifetime adversity (experienced at a moderate level rather than no adversity or nonextreme high adversity) is associated with resilient responses to controlled stressors and is considered beneficial for achieving optimal well-being.^47^ Newer measures capturing the cumulative effect of a range of stressful events (ie, lifetime adversity rather than recent or childhood experiences alone) may better account for the variation in resiliency over time.^36^ A study by Tiet et al., shows youth with higher levels of adverse events were able to express greater resilience if they had a higher IQ, better family functioning, closer parental supervision and monitoring, greater numbers of adults in the household, and higher educational aspirations.^3^ Preferred social factors such as an extended network of family and friends complementing one's parental support may increase the likelihood of developing resilience and stimulating a sense of direction and hope. Future studies could better account for such contextual factors within analyses and assess the mediating/moderating effects of resiliency between stressful life events or unhelpful thoughts with level of incapability.^48^ Greater control, agency, and confidence in managing one's health may have substantial impact on the extent to which stressful life events associated with level of incapability.

### Association Between Pain Intensity and Stressful Life Events

Increased pain intensity was associated more with unhelpful thoughts than either DLEs or ACEs, suggesting ameliorating unhelpful thinking is an important factor in accommodating pain. A recent study found that in patients with PTSD-like symptoms (such as intrusive thoughts and distress) following a stressful life event, pain experience was independent of sustained trauma.^49^ This aligns with our results showing that the unhelpful thoughts have the strongest association with pain experience, regardless of the recent event. As detailed above, future studies might assess the impact of resiliency on the relationship between ACEs, DLEs, and pain intensity as well as opening lines of inquiry that account for different contextual factors, such as social unmet needs, in the relationship between stressful life events and pain experiences in patients seeking musculoskeletal care.

A body of evidence also suggests that ACEs may be associated with seeking care for persistent pain and the activation of physiological (neural, endocrine, immune, and inflammatory) pathways.^4,50^ Several authors have expressed exposure to stressful life events during youth may impair endogenous pain inhibition and heighten central sensitization thereby increasing future pain sensitivity and risk of experiencing painful events.^4,50,51^ Research to elucidate these biological mechanisms could continue in parallel with studies designed to uncover the psychosocial factors that may be driving the multifactorial relationships between stressful life events and symptom intensity.^7,52^

## Conclusions

The finding of an association between unhelpful thoughts and magnitude of incapability and pain intensity among people in musculoskeletal specialty care, but not stressful life events, suggests that unhelpful thoughts are the key factor. There is some evidence that other aspects of mental health (e.g., resiliency) and social health (e.g., unmet needs) might act as mediators or moderators of the association between patient-reported outcomes with unhelpful thoughts. Strategies that help focus our attention toward nurturing the healthiest possible thoughts and behaviors regarding symptoms may ameliorate the impact of stressful life events to achieve improved incapability and response to pain.

## Appendix

**Appendix 1.** Multicollinearity Between Variables.

	Promis Anxiety Cat	Promis Depression Cat	Adverse Childhood Events	Difficult Life Events	NPTQ-4
PROMIS anxiety CAT	1.0	***−***	***−***	***−***	***−***
PROMIS depression CAT	0.80	1.0	−	−	−
Adverse childhood events	0.38	0.30	1.0	−	−
Difficult life events	0.48	0.42	0.40	1.0	−
NPTQ-4	0.51	0.47	0.27	0.37	1.0

The mental health measures NPTQ, PROMIS anxiety CAT, and PROMIS depression CAT are correlated and might explain the problems observed in the MV model when all mental health variables are included.
